# Analysis of Cardiac Events and the Subsequent Impact for Geriatric Patients Undergoing Hip Fracture Surgeries

**DOI:** 10.3390/jcm12165276

**Published:** 2023-08-14

**Authors:** Ting-Cheng Chao, Hsin-Pai Lee, Jung-Chou Wu, Chien-Jen Hsu

**Affiliations:** 1 Department of Orthopaedics, Kaohsiung Veterans General Hospital, Kaohsiung 81362, Taiwan; e871025871220@gmail.com; 2 Department of Orthopaedic Surgery, Ping-Tung Christian Hospital, Pingtung 90059, Taiwan; hplee0929@gmail.com (H.-P.L.); wujcdd@ms10.hinet.net (J.-C.W.)

**Keywords:** hip fracture, cardiac event, echocardiography, in-hospital mortality

## Abstract

Perioperative complications, particularly cardiac events, compromised surgical outcomes for geriatric patients. This retrospective study intended to investigate the occurrence and subsequent impact of cardiac events for geriatric patients undergoing hip fracture surgeries. We collected 607 patients undergoing hip fracture surgeries from January 2017 to December 2022 that received transthoracic echocardiography (TTE) pre-operatively to screen for cardiac abnormalities. Except for demographic characteristics, the researchers recorded fracture type, surgical method, American Society of Anesthesiologists (ASA) class, anesthesia type, perioperative cardiac events, and in-hospital mortality. Statistical analysis was performed using SPSS 22.0 statistics software. Throughout the whole course of the study, 16 postoperative cardiac events occurred. The cardiac events included ten arrhythmias, three acute myocardial infarctions, two heart failures, and one sudden death. Notably, 12 of 16 patients with cardiac events presented with abnormal findings on TTE, except 15 of them had a history of cardiac disease. This study disclosed 93.7% of cardiac events developed in patients with a history of cardiovascular disease. Among patients that experienced cardiac events, 75% of patients had abnormal echocardiographic findings. Pre-operative transthoracic echocardiography deserves a recommendation for geriatric patients with histories of cardiac diseases undergoing hip fracture surgeries to detect the risk of developing cardiac events earlier.

## 1. Introduction

Among elderly patients, hip fracture is a common injury that causes a major public health concern due to the massive consumption of medical resources [1]. Except for considerable expenditure for surgical treatment, concomitant co-morbidities usually add cost for medical care. Furthermore, the subsequent progression of co-morbidities significantly increases the financial burden on the caregiver or their family due to loss of independence [2].

Currently, operative treatment with internal fixation or arthroplasty remains the first priority of treatment choices in order to maintain or restore previous daily activities as soon as possible. Since the average age of patients sustaining hip fractures was quite old, the underlying diseases became an unfavorable factor in superimposing either anesthetic or operative risk throughout the whole course of surgical treatment [3]. Among the common underlying diseases, cardiovascular disease is supposed to be the major one to result in in-hospital mortality, even a late occurrence of mortality caused by a poorly treated cardiac event.

Although, previous studies reported that delayed surgery is associated with worse outcomes after hip fracture surgery [4,5,6]. Therefore, early operative treatment within 24–48 h is suggested to minimize the potential morbidity/mortality associated with deterioration of co-morbidities [6,7]. Nonetheless, recent studies disclosed that cardiovascular events are one of the most common complications following hip fracture surgery [8]. In addition, previous studies indicated that the principal causes of in-hospital death after hip fracture were cardiac failure and myocardial infarction, occurring early after the fracture [9,10]. Because of that, cardiovascular disease could cause impairment of cardiac pumping functions and myocardial fibrosis to increase the risk of heart dysfunction. Patients with cardiovascular diseases were observed to develop more hemodynamic instability intra-operatively that were, therefore, unfavorably classified as a higher American Society of Anesthesiologists (ASA) classification [11]. Moreover, cardiovascular disease is closely related to other chronic medical diseases, like cerebrovascular disease, diabetes, and kidney disease, that are commonly concomitant with the old age of hip fracture patients [12]. Considering the impact of cardiac events, pre-operative risk assessment for potential cardiac events should not be ignored.

Transthoracic echocardiography (TTE) is a non-invasive but reliable modality that can evaluate cardiac function and structural abnormalities and provide important information that has been suggested as a tool to identify patients at risk of perioperative cardiac complications. According to cardiac risk assessment, it could be more efficient to adjust perioperative medication, anesthesia management, and perioperative monitoring during surgery. However, the arrangement of transthoracic echocardiography (TTE) might delay the operation if the patients were not sent to the hospital within regular hospital hours [13,14]. On the other hand, the common strategy for treating patients with hip fractures and concomitant underlying diseases was to conduct the operations as early as possible to reduce the potential morbidity/mortality. In addition, early operations have been reported to facilitate the initiation of rehabilitation for achieving better functional outcomes in addition to shortening hospital stays [13,15,16]. We, therefore, intended to investigate the finding of pre-operative transthoracic echocardiography (TTE) and subsequent development of cardiac events to assess the necessity of arranging pre-operative TTE for geriatric patients undergoing hip surgeries.

## 2. Materials and Methods

### 2.1. Study Population and Data Collection

We collected data on hip fracture patients treated surgically between January 2017 and December 2022 by reviewing the medical records of our hospital with permission from the Institutional Review Board (IRB No: KSVGH23-CT6-04) for this study. We excluded patients with multiple traumas, pathological fractures, and periprosthetic fractures. The collected data were classified as pre-operative characteristics, including age, gender, fracture pattern, American Society of Anesthesiologists (ASA) classification, and finding of transthoracic echocardiography; operative course, including pre-operative waiting time, anesthetic way, surgery type, and operative time; and postoperative follow-up, including postoperative cardiac events, in-hospital mortality, and length of hospital stay (Table 1). 

The transthoracic echocardiography was usually performed within 36 h after hospitalization in order to prevent the extension of waiting time for operation. The abnormality of echocardiography was defined if the demonstration of echocardiography contained one of the following findings: moderate to severe valvular stenosis, regurgitation (aortic, mitral, and tricuspid valve), left ventricle ejection fraction (LVEF) < 50%, congestive heart failure (CHF), or systolic pulmonary artery pressure (PAP) > 55 mnhg.

### 2.2. Definition of Cardiac Event and Cardiac Death

Cardiac events were determined once the following condition was confirmed, including acute myocardial infarction, myocardial ischemia attack, congestive heart failure, and major arrhythmia (atrial fibrillation with rapid ventricular response or ventricular tachycardia). Moreover, primary cardiac death was defined if no other non-cardiovascular complications could be attributed in case of mortality after cardiac event.

### 2.3. Statistical Analysis

The statistical analyses were conducted, including continuous variables expressed as mean ± standard deviation (SD) with comparison by using Student’s *t*-test and categorical variables expressed as percentages with comparison by using the Chi-squared test or Fisher’s exact test. We focused on the analyses of the correlation between cardiac events and results of echocardiography, patient’s history of heart disease, ASA classification, and mortality. Statistical analyses were performed by using SPSS 22.0 statistics software and considered *p* value less than 0.05 as statistically significant.

## 3. Results

By retrospectively reviewing the medical records of our hospital, 827 patients who underwent hip fracture surgeries from January 2017 to December 2022 were investigated. Among 827 patients, 607 patients ever undertook transthoracic echocardiography (TTE) pre-operatively (Table 1).

The demographic characteristics of 607 patients that met the inclusion criteria are displayed in Table 1. The gender distribution revealed that most patients were female almost as twice as male. The average age was extremely old, with a mean age of 81.84 ± 7.49 years. Our policy for treating hip fractures was an internal fixation with the proximal femur nail for intertrochanteric fractures and bipolar hemi-arthroplasty for femoral neck fractures. 

Among 607 patients, 106 patients were traced to have a history of cardiovascular disease (106/607 = 17.5%). Nonetheless, among nine deaths, seven patients had a history of cardiovascular disease (7/9 = 77.8%). Moreover, 142 patients (142/607 = 23.4%) were found to have an abnormality on transthoracic echocardiography. However, among nine deaths, five patients had positive transthoracic echocardiography (5/9 = 55.6%). Previous history of cardiovascular disease and positive transthoracic echocardiography should not be overlooked. Pre-operative anesthetic risk assessment showed nearly 90% of patients comprising ASA III and IV. It implied a higher anesthetic risk to which more attention should be paid in order to prevent exaggeration of co-morbidity, particularly cardiac events. The average pre-operative waiting time is 1.15 days even after completion of transthoracic echocardiography. It was supposed to be acceptable in clinical practice in consideration of better preparation for operation. 

Throughout the whole course of the study, 16 postoperative cardiac events occurred. The cardiac events included ten arrhythmias, three acute myocardial infarctions, two heart failures, and one sudden death. Notably, among 16 patients sustaining cardiac events, 15 patients had histories of cardiac diseases. Moreover, 12 patients had abnormal echocardiographic findings (Table 2).

Among nine mortalities, five patients were classified as cardiac etiology. All five patients ever had histories of cardiac diseases. Four of them demonstrated abnormal echocardiographic findings pre-operatively (Table 3). Analysis for comparing mortality and survived patients (Table 4), unfavorable factors in the mortality group, included a higher percentage of ASA III and IV and occurrence of cardiac events. However, only the occurrence of cardiac events demonstrated statistically significant.

## 4. Discussion

In previous studies, there was no consensus in conducting pre-operative echocardiography for elderly patients with hip fractures routinely [15,17,18]. However, hip fractures were reported to be associated with an in-hospital mortality rate of 7–14%, even reaching up to 36% within 1 year of surgery [9,10,19] over the past decades. Not to mention recent studies also indicated that cardiovascular events were one of the most common complications following hip fracture surgery [20]. In this study, we did disclose that 5 of 16 patients sustaining cardiac events developed mortalities. The considerable mortality rate of over 30% deserves more attention to establish a policy for early detection of developing cardiac events. Therefore, transthoracic echocardiography is supposed to be a rational modality as a pre-operative assessment without invasiveness. However, there is no consensus on whether it was worth extending the pre-operative waiting time for echocardiography or not. A Swedish study on 59,675 patients reported an association between waiting time to surgery and mortality only for patients of ASA III or IV undergoing hip fracture surgeries. Another study suggested unnecessary echocardiography should be reasonably avoided before hip fracture surgery [14,21]. In this study, the average pre-operative waiting time was 1.15 days, even after echocardiography. If the patients had a previous history of cardiovascular diseases, a waiting time of about one day for echocardiography should be acceptable in consideration of preventing cardiac events. Pincus et al. also reported that a waiting time of 24 h represented an acceptable threshold [22].

Regarding ASA classification for assessing anesthetic risk, we noticed patients of ASA III and IV comprised over 90% of the patient population. American Society of Anesthesiologists (ASA) class was a comprehensive assessment of anesthetic risk with consideration of general conditions and co-morbidities. The data of this study implied a higher anesthetic risk for geriatric patients with hip fractures than the ordinary population. Previous research also indicated that patients with higher ASA anesthesia classification had relatively elevated postoperative mortality rates [2,23]. However, we did not conduct a detailed statistical analysis according to the different ASA classifications due to the disproportionate distribution of case numbers among the four classes. Intra-operative hemodynamic instability was another unfavorable factor that should be considered for causing cardiac events. Nonetheless, either internal fixation with proximal femur nailing or bipolar hemi-arthroplasty took only about one hour to operate. During the limited operative course, no countable event of hemodynamic instability was recorded on account of prompt management of hemodynamic change. Consequently, we did not explore the anesthetic records except the anesthetic way and ASA classification. 

Positive findings on echocardiography might proceed ahead of cardiac events, particularly affecting geriatric patients older than 65 years old. Therefore, significant findings on echocardiography should be defined with a clear principle in order to achieve a consensus among medical staff. In general, the cardiac events included acute cardiac infarction, heart failure, moderate to severe valvular stenosis or regurgitation, and arrhythmia. Sixteen patients developed cardiac events, and twelve of them (75%) had obvious cardiac abnormalities on echocardiography [10,14]. Several studies indicated that moderate to severe aortic stenosis caused by calcification played a critical role in postoperative cardiac complications [23,24]. Another previous study reported that both aortic stenosis and LVEF < 50% related to arrhythmia or heart failure were significant factors in causing cardiac events [19,25]. The above-mentioned findings of echocardiography, including moderate to severe valvular stenosis or regurgitation, LVEF < 50%, congestive heart failure, and pulmonary arterial pressure > 55 mnhg, were therefore considered significant. Even though only 16 cardiac events occurred among 607 patients having TTE, transthoracic echocardiography might be considered for patients with histories of cardiac diseases.

In addition, the in-hospital mortality rate in our study was 1.5%, which was lower than in previous studies [26,27]. Among nine mortalities, seven patients had a history of cardiovascular disease. As a consequence, more attention should be paid to those patients with histories of cardiovascular disease, even though a lower mortality rate was noticed than in other reports. Pre-operative echocardiography also revealed positive findings for five patients of nine mortalities (55.6%). Eventually, five patients sustaining cardiac events developed mortalities, demonstrating a significant association between 16 cardiac events and five in-hospital mortalities. Among nine deaths, five patients died of cardiac event-related etiology. Four patients of them were disclosed to have a concomitant history of cardiovascular disease and positive transthoracic echocardiography (TEE) (Table 3). This study disclosed an association not only between cardiac events and previous history of cardiovascular disease but also between abnormal findings in echocardiography and in-hospital mortality rate. Consequently, the positive finding of pre-operative TEE might imply an ongoing cardiovascular condition. The medical staff could be reminded of the prevention of the progression of cardiovascular events in advance.

There were several limitations in this study that needed to be clarified. First, this study only recorded ASA classification as an anesthetic risk assessment without individualized investigation of other co-morbidities that might alter the risk assessment. Since the ASA classification was a comprehensive assessment, it might attenuate the impact of lacking investigation of co-morbidities. Second, we could not provide the actual reason for the choice of anesthetic way that might cause different risks of developing cardiac events. This was a retrospective study collecting information from medical records where the researchers did not join every operative decision. However, the operation time was comparably short, about one hour either by general anesthesia or by spinal anesthesia. The influence of the anesthetic way was supposed to be limited. Third, the management of cardiac events was not mentioned in this study. The authors did not take part in the clinical care of these patients personally. We, therefore, could not discuss the outcomes of different management of cardiac events. If a further study is initiated, we will amend the part of management and treatment for cardiac events.

## 5. Conclusions

A high association was disclosed between the onset of cardiac events and previous history of cardiovascular diseases in this study. Furthermore, positive findings on echocardiography suggested a high probability of developing cardiac events. Seven patients of nine mortalities did demonstrate positive findings on echocardiography.

Pre-operative transthoracic echocardiography examinations may detect potential functional abnormalities of the heart, especially for patients with a history of heart disease or related symptoms. Consequently, early adjustment of medications and treatment during anesthesia became beneficial to decrease the occurrence of cardiac events for geriatric patients undergoing hip fracture surgeries.

## Figures and Tables

**Table 1:** Demographic characteristics.

Patient n = 827		With Echocardiography n = 607		No Echocardiography n = 220		*p*-Value
Age (mean ± SD years)		81.84 ± 7.49		78.07 ± 8.02		*p* < 0.01
Gender (Male/Female)		200/407		71/149		0.855
Fracture type						
Femoral neck		271	(44.6%)	95	(43.2%)	0.708
Intertrochanteric		336	(55.4%)	125	(56.8%)	
Surgery type						
Hemi-arthroplasty		271	(44.6%)	95	(43.2%)	0.708
PFN		336	(55.4%)	125	(56.8%)	
ASA classification						
I		6	(0.98%)	1	(0.45%)	0.066
II		67	(11.0%)	37	(16.8%)	
III		411	(67.7%)	149	(67.7%)	
IV		123	(20.3%)	33	(15.0%)	
Anesthesia						
General		105	(17.3%)	44	(20.0%)	0.372
Spinal		502	(82.7%)	176	(80.0%)	
Pre-operative waiting time (day)		1.15		0.67		*p* < 0.01
Operation time (minute)		77.1		69.7		0.505
Cardiac events		16	(2.64%)	2	(0.91%)	0.133
In-hospital mortality		9	(1.48%)	2	(0.91%)	0.525
Hospital stay (day)		7.61		6.73		*p* < 0.01
SD: standard deviation		PFN: proximal femoral nail				
ASA classification: The American Society of Anesthesiologists (ASA) physical status classification						

**Table 2:** Analysis of association between cardiac event and predisposing factors.

Cardiac Event	History of CV Disease	TTE Finding	Mortality
1. Af with RVR	CAD	Negative	
2. Af with RVR	Af with RVR	Severe TR	
3. Myocardial ischemia attack	CAD	Moderate TR, pul HTN	
4. Myocardial ischemia attack	CAD	Severe TR	
5. Af with RVR	Af with RVR	Moderate TR Pericardial effusion	
6. Af with RVR	Af with RVR	Moderate TR, pul HTN	
7. CHF	None	Negative	
8. Af with RVR	CHF	Severe TR pul HTN	
9. Af	Af	Negative	
10. Af with RVR	CAD	Moderate MR, TR	
11. Af	Af	Moderate AR, TR	
12. CHF	CHF	EF37%, pericardial effusion	+
13. AMI	CAD	Moderate TR	+
14. Af with RVR	CAD	Moderate AS	+
15. Sudden death	CAD	Negative	+
16. Sudden death	Moderate AR	Moderate TR	+

CV: cardiovascular, Af: atrial fibrillation, CAD: coronary artery disease, CHF: congestive heart failure, RVR: rapid ventricular response, AMI: acute myocardial infarction, TR: tricuspid regurgitation, MR: mitral regurgitation, AR: aortic regurgitation, AS: aortic stenosis, EF: ejection fraction, HTN: hypertension.

**Table 3:** Analysis of in-hospital mortality and its association with predisposing factors.

Mortality	With History of CV Disease	TTE Positive	Cause of Death
1	+	+	Af with RVR
2	+		AMI
3	+	+	COPD with AE, pneumothorax, Cor pulmonale
4	+	+	AMI
5	+	+	AMI
6	+	+	UGI bleeding
7	+		Urosepsis
8			UGI bleeding, pneumonia
9			Pneumonia

CV: cardiovascular, Af: atrial fibrillation, RVR: rapid ventricular response, AMI: acute myocardial infarction, AE: acute exacerbation.

**Table 4:** Comparison between mortality and survival for risk factors.

Parameters n = 607	Mortality n = 9	Survival n = 598	*p*-Value
Age	84.33 ± 4.74	81.80 ± 7.52	0.314
M/F	5/4	195/403	
Fracture type			
Femoral neck	4 (44.4%)	267 (44.6%)	0.146
Trochanteric	5 (55.6%)	331 (55.4%)	
Surgery type			
Hemi-arthroplasty	4 (44.4%)	267 (44.6%)	0.990
Proximal femoral nail	5 (55.6%)	331 (55.4%)	
Anti-rotation			
ASA class			
ASA I	0	6 (1.0%)	
ASA II	0	67 (11.2%)	0.059
ASA III	4 (44.4%)	407 (68.0%)	
ASA IV	5 (55.6%)	118 (19.7%)	
Anesthesiology type			
General anesthesia	2 (22.2%)	103 (17.2%)	0.694
Spinal anesthesia	7 (77.8%)	495 (82.8%)	
Pre-op wait time (day)	1.22	1.30	0.862
Hospital stay (day)	5.00	7.91	0.018 *
OP time (min)	73.6	77.8	0.944
Cardiac events	5	11	*p* < 0.01 *

“*” means statistically significant.

## Data Availability

The research data is not publicly available due to privacy or ethical consideration. Once this manuscript is accepted for publication, delinked data without personal privacy could be provided upon reasonable request.
